# Laboratory toxicological assessment of ozone exposure on terrestrial snail *Theba pisana* and its impact on histopathological alterations

**DOI:** 10.1038/s41598-026-44106-2

**Published:** 2026-03-31

**Authors:** Khaled H. Metwaly, Ruwaida Elhanbaly, Mohamed A. Awad, Mohammed E. Gad, Hesham A. M. Ibrahim

**Affiliations:** 1 Center of Plasma Technology, Al-Azhar University, Cairo, 11884 Egypt; 2 Department of Anatomy and Embryology, Faculty of Veterinary Medicine, Assiut University, Assiut, 71515 Egypt; 3 Department of Zoology and Entomology, Faculty of Science, Al-Azhar University, Nasr City, 11884 Cairo Egypt; 4 Department of Agricultural Zoology and Nematology, Faculty of Agriculture, Assiut Branch, Al-Azhar University, Assiut, 71524 Egypt

**Keywords:** *Theba pisana*, ozone exposure, histopathology, terrestrial snail, mortality rate, weight loss, Diseases, Environmental sciences, Zoology

## Abstract

This laboratory study assessed the toxicological effects of ozone gas exposure on the terrestrial snail *Theba pisana* (Müller, 1774). Adult snails were exposed to different ozone concentrations for 30 min to evaluate mortality, changes in body weight, and histopathological alterations. Results showed that mortality and weight loss increased with higher ozone concentrations and longer exposure times, reaching 16.67, 20.00 and 53.33% mortality and 5.21, 7.34 and 8.43% weight loss percentages at 250, 500 and 1000 ppmv after 24 h post-exposure. Histopathological analysis revealed progressive damage in the digestive gland, ranging from mild destruction to complete rupture, along with degeneration and necrosis in foot tissues. These findings demonstrate that ozone induces pronounced toxic effects on *T. pisana* under laboratory conditions and provide baseline information on its biological and histopathological impact. However, further studies are required to evaluate lower, environmentally relevant concentrations, non-target effects, and potential practical applications.

## Introduction

 Several species of terrestrial gastropods, including snails and slugs, are recognized as economically important animals in agroecosystems due to their feeding behavior, nocturnal activity, and high reproductive capacity, which collectively contribute to crop damage and economic losses^1–3^. They are members of the broad taxonomic group of invertebrates within the phylum Mollusca. The key distinction between slugs and snails lies in the fact that snails possess external shells and can retract their soft bodies into them when threatened^4^. Land snails possess several protective mechanisms, the most important being the shell and mucous secretions. They can retract their soft bodies into the shell when threatened by predators or during periods of drought and high temperatures. Gastropods secrete a mucus trail that serves multiple functions, including lubrication, adhesion, moisture retention, and defense^5–7^. It reduces friction, aids in retaining moisture, and provides resistance against microbial infection^8^. Among the many other functions performed by the mucus trail, including the provision of mechanisms for path retracing and for trail-following in order to locate a mate of the same species^9^. The white garden snail, *Theba pisana* (Müller, 1774), is widely distributed in many regions of the world and is considered a problematic species in agricultural and horticultural systems, particularly in areas where it has been introduced beyond its native range and has become an invasive species^10–15^. Its ecological success is attributed to its broad host range, high reproductive potential, and ability to tolerate diverse environmental conditions. Ozone (O₃) is a highly reactive oxidizing agent that decomposes rapidly into molecular oxygen, with a short half-life ranging from approximately 20 to 50 min under ambient conditions^16^. Due to its strong oxidative properties, ozone has been extensively studied for its biological effects on a wide range of organisms, including insects associated with stored products, primarily grain insects, microorganisms^17,18^. Ozone has also been employed as a disinfectant and sanitizer to degrade pesticide residues and organic and inorganic contaminants, as well as to control microorganisms and insects^19^. Applying ozone to stored products is effective in decomposing pesticide residues and controlling insects, fungi, and bacteria. Therefore, ozone gas was selected for evaluation against *T. pisana* due to its strong oxidative and toxic properties, which may induce physiological stress in terrestrial snails^20^. Histological investigations serve as crucial indicators for elucidating the toxic effects induced by various compounds in terrestrial snails^21–27^. Structural alterations in key organs, such as the digestive gland and foot, are commonly used as sensitive indicators of toxic exposure and physiological impairment in gastropods. Despite the documented biological effects of ozone on various organisms, information regarding its toxicological impact on terrestrial snails, particularly *T. pisana*, remains limited. Therefore, the present study aimed to evaluate the toxic effects of controlled ozone exposure on adult *T. pisana* under laboratory conditions, with particular emphasis on mortality, changes in body weight, and histopathological alterations in the digestive gland and foot tissues. This work provides baseline toxicological data that contribute to a better understanding of ozone-induced biological responses in terrestrial gastropods.

## Results

### Toxicity of gaseous ozone towards the terrestrial snail *T. pisana*

Table 1 presents the mortality of the white garden adult snails *T. pisana* following 30 min of exposure to three laboratory-applied ozone concentrations. Results indicated that mortality rates increase with increasing concentrations and the period after exposure. The highest death rates for land snails, *T. pisana*, were recorded at the highest concentration (1000 ppmv), with values of 26.67, 40.00 and 53.33% after 24 h, 48 h, and 96 h of treatment with ozone gas, respectively. On the other hand, the lowest death rates were recorded for the treated snails at the lowest concentration (250 ppmv), with values of 3.33, 10.00 and 16.67% after 24, 48 and 96 h of treatment for half an hour, respectively. The general mean death rates of the treated snails at concentrations of 250, 500 and 1000 ppmv were 10.00, 15.55 and 40.00%, respectively. Based on probit analysis conducted at 96 h post-exposure, the estimated lethal concentrations (LC values) of ozone for adult *T. pisana* were 192.46, 431.85, and 1060.03 ppmv corresponding to LC₁₀, LC₂₅, and LC₅₀, respectively. It was also noted that when exposed to doses of ozone, this causes the snails to secrete mucus secretions from the snail’s body, and accordingly, weight reduction was estimated for the exposed snails to ozone gas.

**Table 1:** Efficacy of different concentrations of ozone gas against the land snails, *Theba pisana*.

Periods after exposure	% Mortality at indicated concentrations (ppmv)			
	Control group	Experimental groups		
	0	250 ppmv	500 ppmv	1000 ppmv
24 h	0	3.33	10.00	26.67
48 h	0	10.00	16.67	40.00
96 h	0	16.67	20.00	53.33
Mean	0	10.00	15.55	40.00

Each treatment group consisted of 30 adult snails, divided into three replicates of 10 adults each.

Values represent mortality percentages corrected using Abbott’s formula based on the control group.

### Influence of different concentrations on weight loss of *T. pisana*

Ozone exposure induced a clear concentration-dependent increase in weight loss of adult *T. pisana* body (Table 2). The control group (0 ppmv) showed negligible weight reduction (0.01 ± 0.05 g; 0.09 ± 0.58%), reflecting normal physiological variation in untreated snails. In contrast, ozone-treated groups exhibited markedly higher weight loss. The highest values were recorded at 1000 ppmv (Group 4), followed by 500 ppmv (Group 3), while the lowest loss among treated groups was observed at 250 ppmv (Group 2), with mean absolute weight losses of 0.68 ± 0.17, 0.59 ± 0.17 and 0.40 ± 0.06 g, respectively. Similarly, relative weight loss increased from 5.21 ± 0.50% at 250 ppmv to 7.30 ± 2.19% and 8.26 ± 2.08% at 500 and 1000 ppmv, respectively, indicating increased physiological stress with rising ozone concentration. This reduction is likely associated with enhanced mucus secretion and dehydration following ozone exposure. Statistical analysis using one-way ANOVA, including the control group, revealed a significant effect of ozone concentration on weight loss (*P* < 0.05). To further account for the distributional characteristics of the data, a generalized linear model (GLM) with a Gamma distribution and log link function was applied, which confirmed a highly significant treatment effect (Wald χ² = 131.11, df = 3, *p* < 0.001). Both analyses consistently demonstrated a concentration-dependent increase in weight loss, supporting the robustness and reliability of the observed treatment response.

### Effect of ozone on the shell surface appearance of the terrestrial snail *T. pisana*

**Fig. 1:**
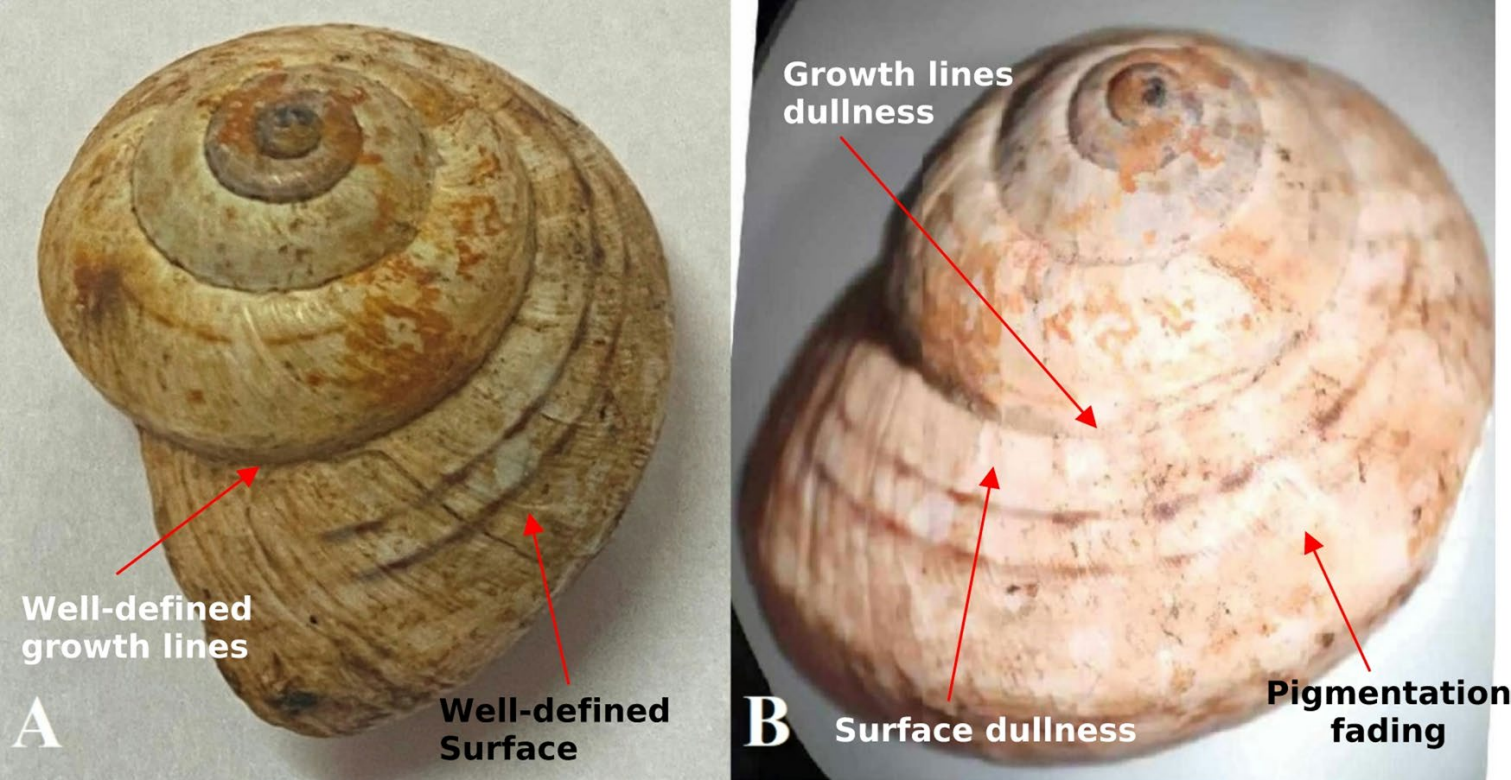
Appearance changes in the shell morphology of the snail *Theba pisana* before and after exposure to ozone gas.

Macroscopic examination revealed noticeable changes in the appearance and surface structure of the outer shell as a result of treatment with ozone gas (Fig. 1). It was found that the outer shell surface before exposure was shiny and the growth lines and mottled brown bands were clear (Fig. 1A), while after exposure to ozone gas, the outer shell surface and growth lines were dull, and the mottled bands were relatively faded (Fig. 1B). In the control specimens, we also noticed the presence of spots and dirt on the outer surface of the shell in (Fig. 1A (before exposure to ozone gas, and these spots are the result of the snail’s movement and its adhesion to the soil surface in the field. But after exposure to ozone gas, these spots disappeared, and the effect of the ozone reached the surface of the shell itself (Fig. 1B).

**Table 2:** Influence of ozone gas application on weight loss of *Theba pisana* adult snails after 24 h post-treatment.

Treatments (Con.)	Initial group weight (g) Mean ± SE	Final group weight (g) Mean ± SE	Weight loss (g) (Mean ± SE)	Weight loss (%) (Mean ± SE)
Group 1 (0 ppmv)	8.02 ± 0.47^a^	7. 98 ± 0.47 ^a^	0.05 ± 0.01^b^	0.58 ± 0.09^b^
Group 2 (250 ppmv)	7.52 ± 0.52 ^a^	7.13 ± 0.46 ^a^	0.40 ± 0.06^ab^	5.21 **±** 0.50^ab^
Group 3 (500 ppmv)	8.04 ± 0.17 ^a^	7.45 ± 0.23 ^a^	0.59 ± 0.17^ab^	7.30 ± 2.19^ab^
Group 4 (1000 ppmv)	8.17 ± 0.19 ^a^	7.50 ± 0.22 ^a^	0.68 ± 0.17^a^	8.26 ± 2.08^a^

Each treatment group consisted of 30 adult snails, divided into three replicates of 10 adults each.

Mean values within a column sharing the same letter are not significantly different (*P* < 0.05).

GLM analysis (Gamma distribution with log link) showed a significant effect of treatment on weight loss (Wald χ² = 131.11, df = 3, *p* < 0.001).

**Fig. 2:**
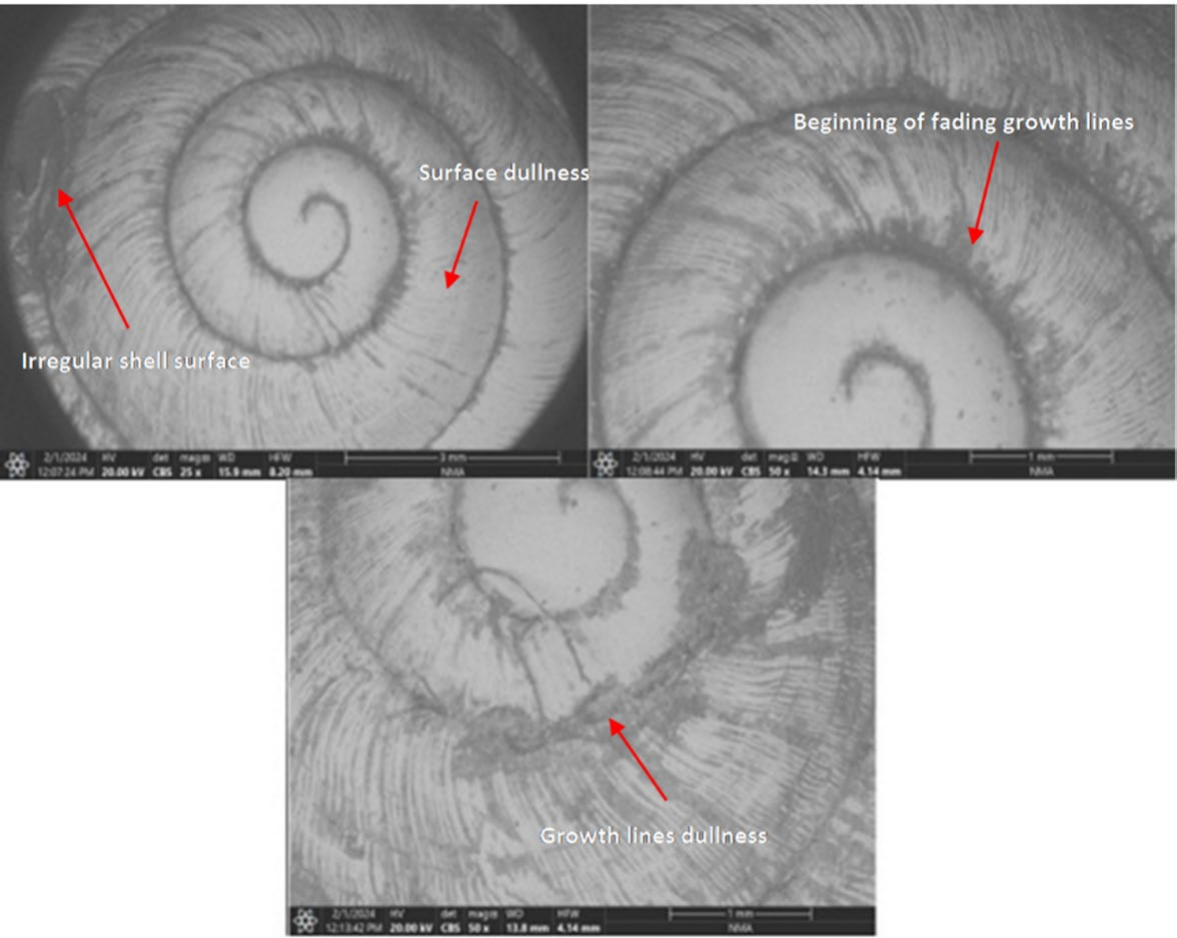
Scanning electron micrographs of the terrestrial snail *T. pisana* at the 500 and 1000 ppmv and 25X, 50X magnification.

**Fig. 3:**
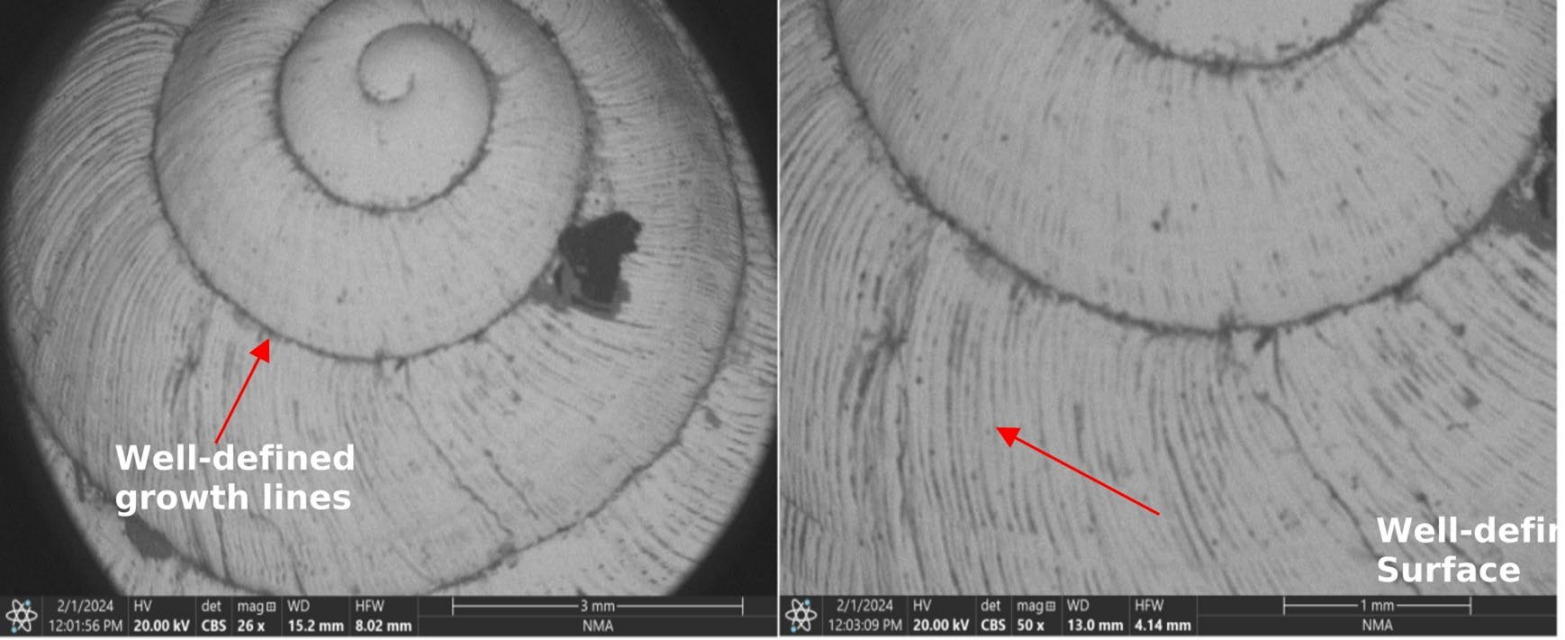
Scanning electron micrographs of control to the terrestrial snail *Theba pisana* at the 26X and 50 X magnifications.

The adult of the terrestrial snail (*T. pisana*) both untreated and treated with different concentrations of ozone, was investigated using a scanning electron microscope (SEM) in Figs. (2, 3) In all treatments, the investigation showed the same changes of surface appearance when using ozone compared to the control treatment, where changes appeared in the morphological features on the dorsal surface of the shell, especially at high concentrations of 500 ppmv and 1000 ppmv ozone concentrations (Fig. 2). On the other hand, no changes appeared on the surface of the snail *T. pisana* in the control treatment (Fig. 3).

These features were confined to the shell surface, accordingly, these observations are described as These features were confined to the shell surface changes in shell appearance and surface microtopography only. This results from the interaction of ozone gas with the surface of the outer shell, which leads to dullness in the surface of the body, with the growth lines in the snail’s outer shell disappearing. In conclusion, after ozone exposure, the shell surface exhibited dullness, fading of pigmentation, and reduced visibility of growth lines. Surface irregularities became more pronounced at higher ozone concentrations (Figs. 2).

### Histopathological observations

#### Digestive gland of snails

**Fig. 4:**
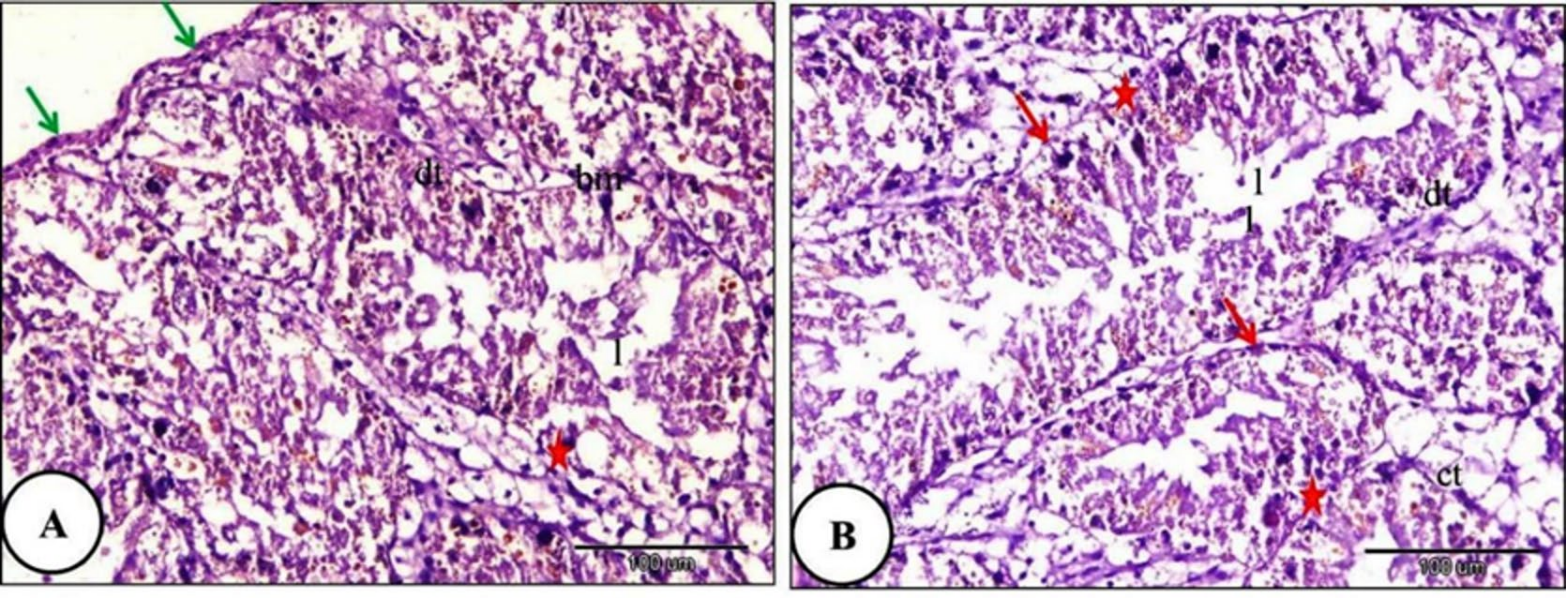
A Photomicrograph illustrating the normal histological structure of the digestive gland in *Theba pisana* (control group): (**A**) the digestive tubule lumen (l), the outer layer (green arrow), and the digestive tubules (dt). (**B**) Intertubular connective tissue (ct) and the basement membrane (red stars), and smooth muscle fibers (red arrows). Stained with Hematoxylin and Eosin.

**Fig. 5:**
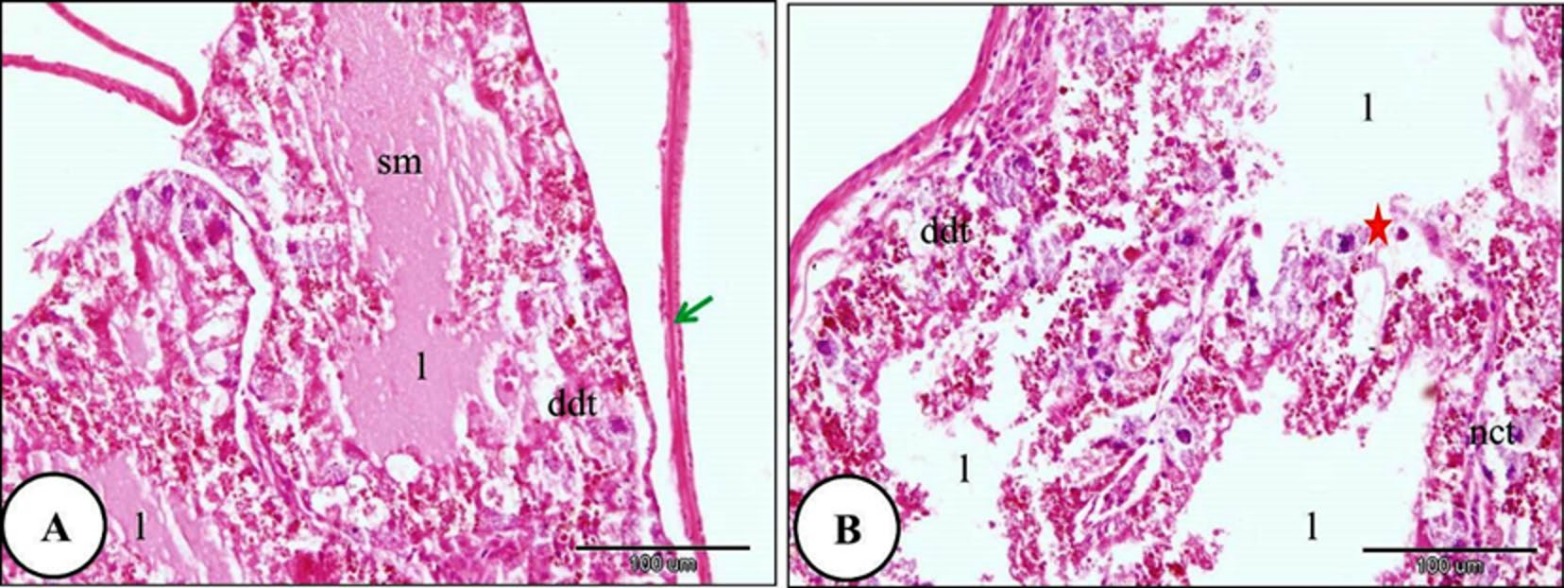
A Photomicrograph of the digestive gland in *Theba pisana* exposed to LC_10_ ozone for 24 h, illustrating: (**A**) detachment of the outer layer surrounding the digestive tubules (green arrow), destruction of the digestive tubules (ddt), and lumen containing secretory materials (sm). (**B**) Necrosis in the connective tissue between tubules (nct) and rupture of the basement membrane (red star) and irregular and branched lumen and more than two tubules connected together with one large lumen. Hematoxylin and Eosin staining.

The histological investigation of the digestive gland of the control group snails showed a bilobed tubulo-acinar structure located in the dorsal region of the snail’s body. It is enclosed by an outer layer of simple columnar cells supported by a basement membrane, followed by an underlying layer of circular muscle fibers. Each lobe contained numerous tubules. The tubules were oval or spherical and were separated by intertubular connective tissue. The lining epithelium of the digestive tubules consisted of columnar cells resting on a thin basement membrane (Fig. 4). In snails exposed to LC₁₀ of ozone for 24 h, slight destruction was observed in some digestive tubules, while others exhibited marked shrinkage and degeneration of the lining epithelial cells. Moreover, the digestive tubules’ lumen width increased as they were filled with secretory contents. Moreover, more than two digestive tubules merged into one large lumen. There was rupture in the basement membrane. In addition, necrosis was observed in the intertubular connective tissue (Fig. 5).

**Fig. 6:**
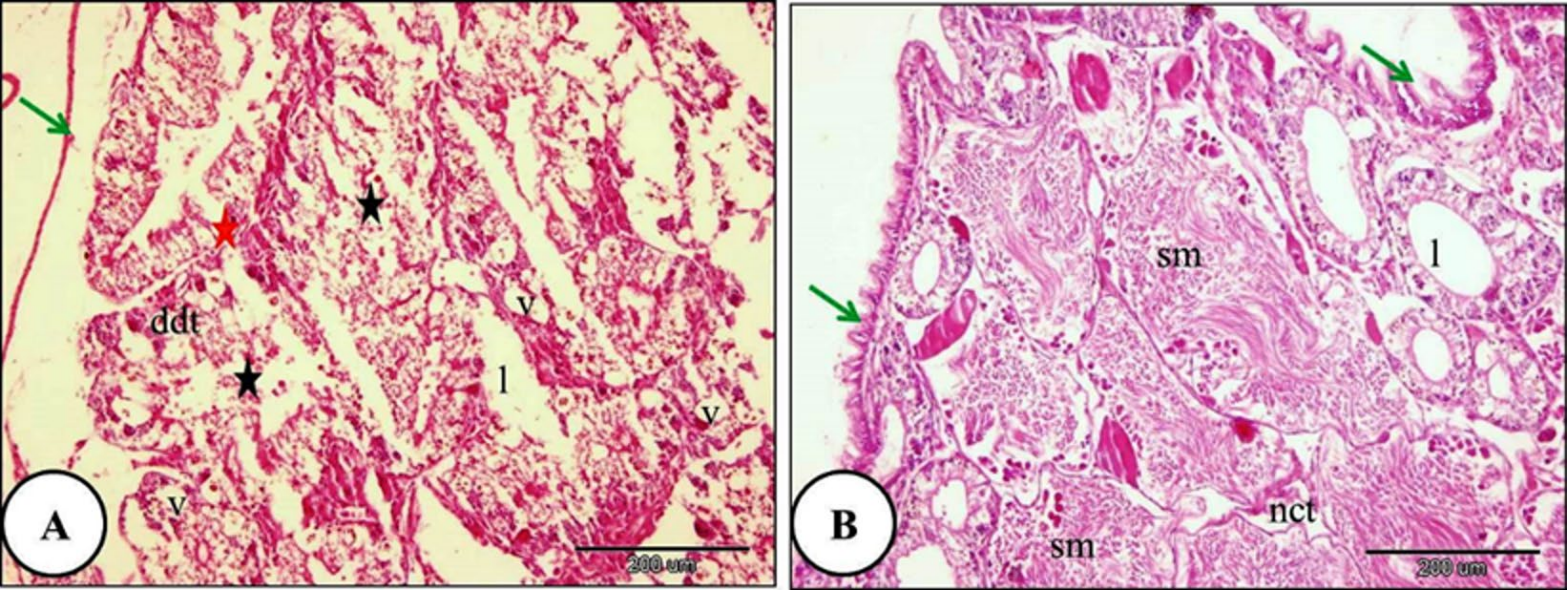
A Photomicrograph of the digestive gland of *Theba pisana* exposed to LC_25_ of ozone for 24 h, showing: (**A**) detachment of the covering the digestive tubule’s outer layer (green arrow), atrophy, necrosis and destruction in the digestive tubules accompanied by basement membrane rupture (red star), detachment of the regions at the top of digestive cells into the tubule lumen (black star), and the majority of the digestive tubules’ epithelium were vacuolated (v). (**B**) Presence of secretory materials in the lumen of the digestive tubules along with necrosis of the intertubular connective tissue (nct). Hematoxylin and Eosin staining.

**Fig. 7:**
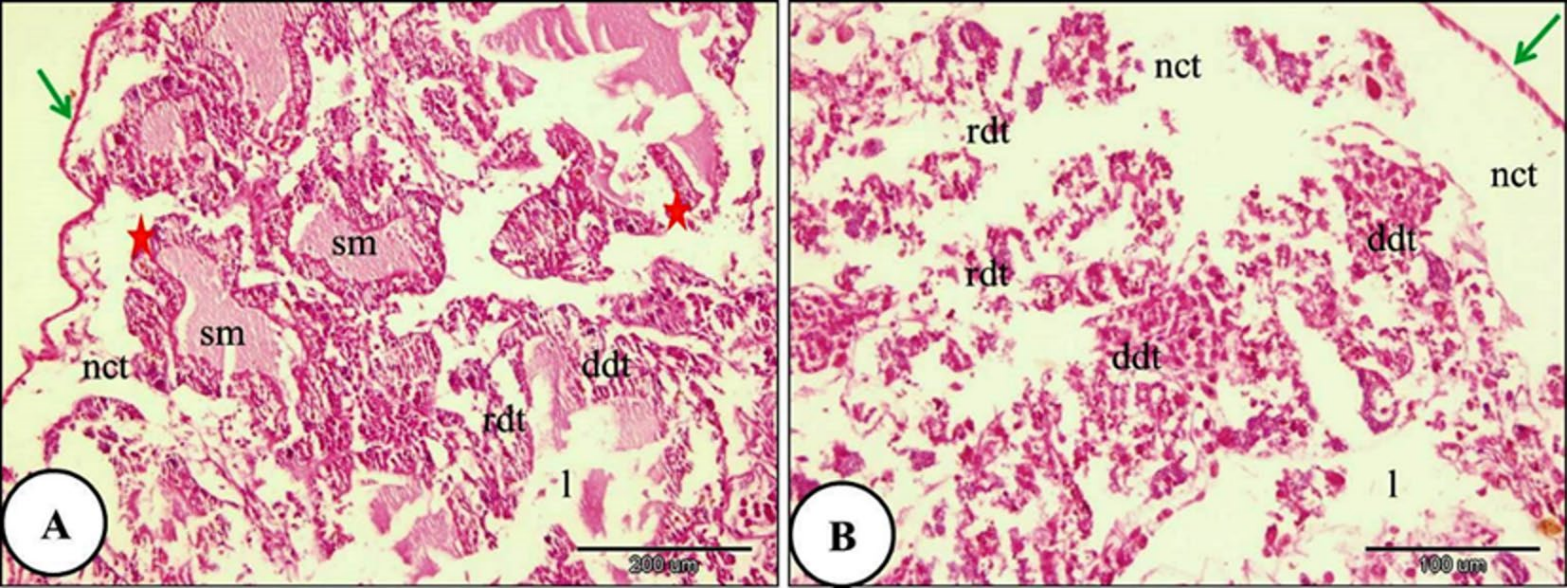
A Photomicrograph of the snails’ digestive gland treated with LC_50_ ozone for 24 h showing; (**A**) The covering the digestive tubule’s outer layer was detached and shrinked (green arrow), atrophy, destruction and necrosis of digestive tubules with basement membrane rupture (red star) and the lumen of digestive tubules were irregular and branched which filled with secretory materials and more than two digestive tubules connected with one large lumen (**B**) The digestive tubules and their constituents lining cells showed complete ruptured (rdt) and the intertubular loose connective tissue showed necrosis (nct).Hematoxylin and Eosin staining.

With increasing ozone concentration, the digestive gland of *Theba pisana* treated with LC₂₅ for 24 h showed severe destruction of some digestive tubules. Destruction of the basement membrane was also observed. Marked disruption of the apical border was observed in most digestive cells, and the detached apical portions formed blebs within the tubule lumen. The secretory materials filled the digestive tubules lumen. Necrosis of the intertubular connective tissue was evident (Fig. 6). Moreover, the digestive gland and their constituents lining cells of *T. pisana* treated with LC_50_ of ozone for 24 h showed complete ruptured and common architecture of digestive gland had disappeared. The lumen width was irregular and branched and full of secretory materials and intertubular connective tissue showed complete necrosis (Fig. 7).

### Foot of snails

**Fig. 8:**
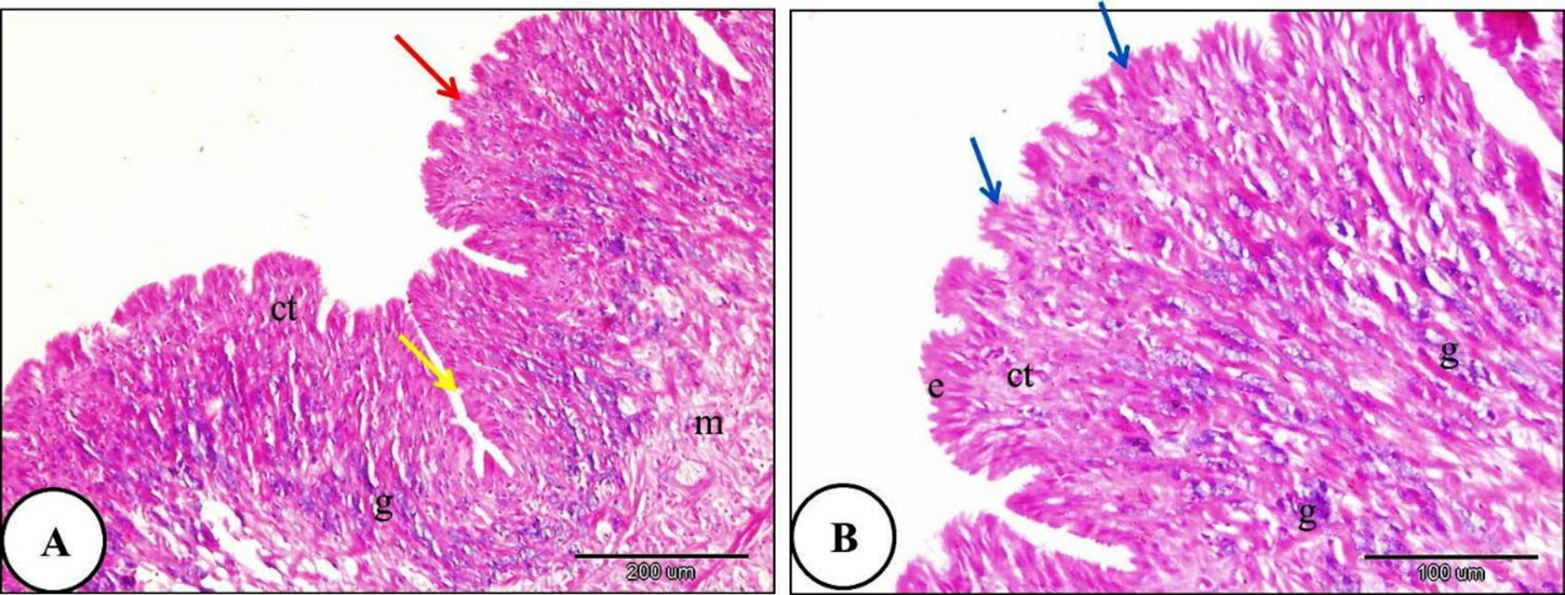
A photomicrograph depicting the general structure of *Theba pisana* foot (control group), illustrating: (**A**) The foot’s side (indicated by yellow arrows) and sole (red arrow), both covered by an epithelial layer (e), the layer of inner connective tissue (ct), muscular tissue (m) and embedded gland (g). (**B**) The foot’s sole, which has pseudostratified columnar epithelium covering it (e), connective tissue layer (ct) housing numerous deeply buried glands (g), along with superficial folds in the epithelial layer of the sole (blue arrows). Hematoxylin and Eosin staining.

**Fig. 9:**
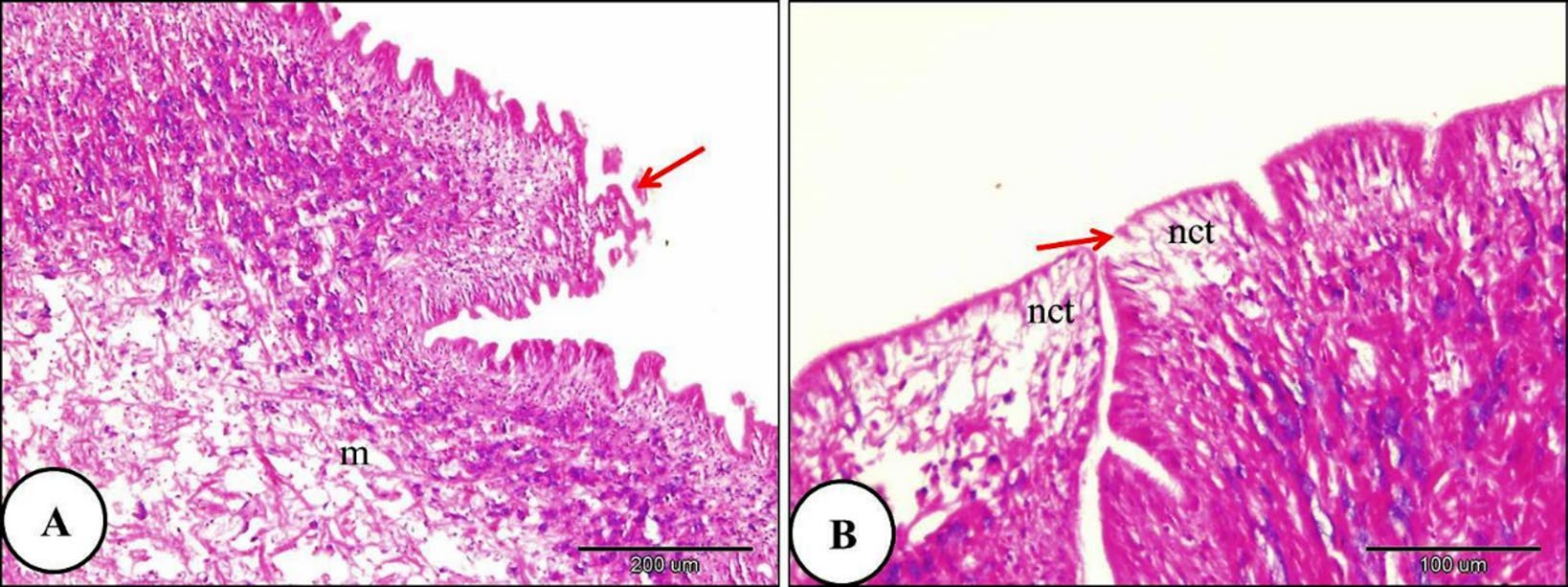
A photomicrograph of the foot of *Theba pisana* exposed to LC_10_ of ozone for 24 h, showing; (**A**) foot-covering epithelium rupture (red arrows). (**B**) Sides of the feet with ruptured epithelial cells (red arrow), destruction of muscular tissue (m), and necrosis of connective tissue (nct). Hematoxylin and Eosin staining.

**Fig. 10:**
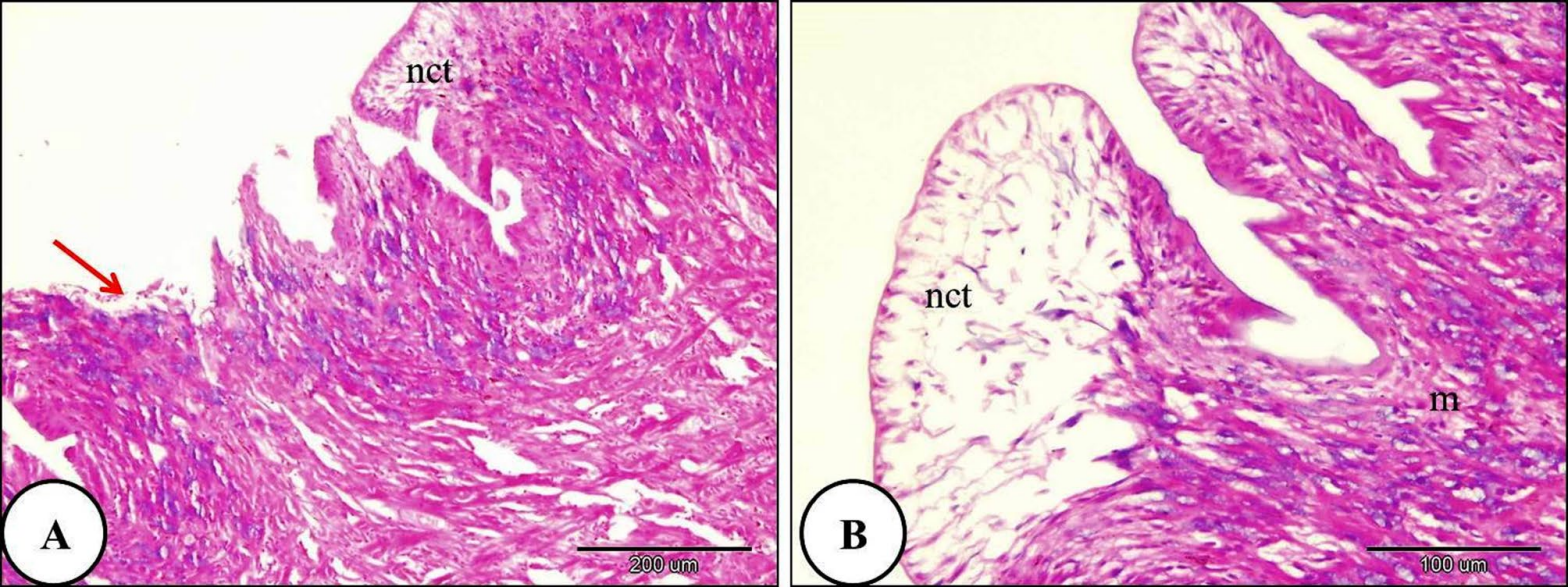
A Photomicrograph of the foot of *Theba pisana* exposed to LC_25_ of ozone for 24 h showing; (**A**) foot-covering epithelium rupture (red arrows). (**B**) muscular tissue destruction (m), and necrosis of connective tissue (nct). Hematoxylin and Eosin staining.

**Fig. 11:**
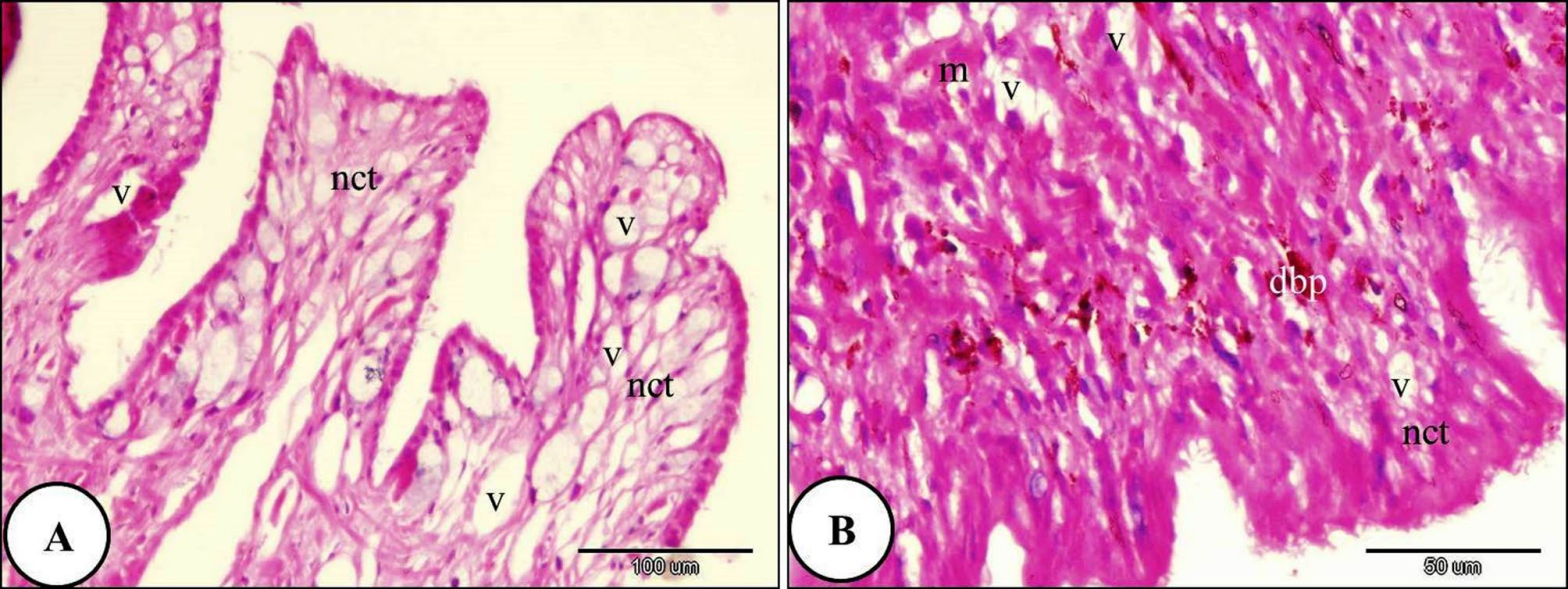
A Photomicrograph of the foot of *Theba pisana* exposed to LC_50_ of ozone for 24 h showing: (**A**) presence of connective tissue vacuoles (v) and necrosis of connective tissue (nct). (**B**) presence of vacuoles in the muscle fiber layer (v) and presence of the connective tissue’s dark brown pigment (dbp). Hematoxylin and Eosin staining.

The histological investigation of the foot of *T. pisana* of the control group revealed internal connective tissue layer is covered by a single exterior layer of epithelium. A layer of pseudostratified columnar epithelium covered the snail foot. The foot consisted of two regions: the sides and the sole. Many glands were shown in the connective tissue layer (Fig. 8). In snails exposed to LC₁₀ of ozone, the epithelial covering of the foot showed rupture. The muscle layer exhibited necrosis and degeneration, and the connective tissue also showed necrotic changes (Fig. 9). Furthermore, the foot of *T. pisana* Snails treated with LC₂₅ of ozone for 24 h showed similar histopathological alterations and deep folds in its sides and necrosis in the layer of connective tissue was more obvious (Fig. 10). The foot of *T. pisana* treated with LC_50_ of ozone necrosis, deformity of muscular tissue and presence of vacuoles as a result of deformation happen in this layer. Also, vacuoles within the connective tissue were observed and appearance of dark pigments (Fig. 11).

## Discussion

Our study demonstrated that ozone gas exhibits a pronounced toxic effect against adult individuals of *T. pisana*, with mortality increasing in a concentration- and time-dependent manner following a 30-minute exposure. This pattern is consistent with the strong oxidative properties of ozone, which can disrupt cellular membranes and interfere with essential physiological processes. The observed increase in mortality with prolonged post-exposure periods suggests that ozone-induced damage may persist beyond the exposure phase, leading to delayed toxic effects. The findings align with the observations made by Hussain^28^, they found that increasing the period of ozone gas exposure led to a gradual increase in mortality rates. Mahmoud et al.^29^ reported that ozone gas was effective against adults and the second and fifth instars of *Trogoderma granarium*, with LC_50_ values of 275.30, 446.75 and 249.76 ppmv after two hours of exposure to ozone gas. Per the work authored by McDonough et al.^30^, it was notated that exposure to 500 ppmv of ozone for a period of 60 min resulted in complete mortality among adult *Plodia interpunctella* specimens.

In another study, Metwally et al.^31^ pointed out that ozone, as a strong oxidizing agent capable of controlling insects and microorganisms, demonstrated effectiveness in eradicating houseflies and pathogenic bacteria. Silva et al.^32^ examined the impact of ozone exposure, at a concentration of 750.8 ppmv, on adult *Rhyzopertha dominica* beetles inhabiting wheat kernels. Their findings indicated that exposure to ozone for durations between 8.69 and 13.08 h resulted in a 50% mortality rate, while exposure durations spanning 11.28 to 18.11 h led to a mortality rate of 95%. In the study, Subramanyam et al.^33^ reported that increased doses of ozone, at levels of 200 and 400 ppmv, were necessary for effectively controlling *R. dominica* adults. Their findings indicate that adult mortality rates following one day of exposure were recorded at 67% and 42%, respectively, for these ozone concentrations. Gad et al.^34^ demonstrated that exposing *Callosobruchus maculatus* adults to 600 ppmv (1.2 g/m^3^) of ozone led to complete mortality after five days at all exposure times (0.5 to 5 h).

The digestive gland of snails carries out many intracellular and extracellular functions. It participates in osmoregulation, excretion, detoxification, and the digestion and absorption of food matrials^35,36^ and any structural alterations may impair these functions. Our study revealed that exposure to different dose of ozone caused significant damage to the digestive gland which of course affecting its fuction. Moreover, ozone exposure also induced severe histopathological changes in the foot of the terrestrial snail *T. pisana* which of course effect on the movement.

Although no previous studies have specifically examined the histopathological effects of ozone on terrestrial snails, comparable tissue alterations have been reported in gastropods exposed to other toxicants. Abd El-Atti et al.^27^ described structural damage in the digestive gland of *T. pisana* following prolonged exposure to chemical compounds, including tubular deformation and cellular vacuolation. Ali^37^ reported extensive degeneration of digestive tubules and connective tissues in *Monacha obstructa* treated with plant extracts, while Ibrahim et al.^38^ documented severe histological alterations in both the digestive gland and foot tissues of treated snails. Similar findings were also reported by Abdl-Kader^39^ and Gaber et al.^26^, who linked tissue destruction to physiological dysfunction and increased mortality. Consistent with previous reports, ozone has been shown to cause deformation and cracking of the insect cuticle^40–42^. While many previous investigations have focused primarily on insects, the present study extends the toxicological relevance of ozone to terrestrial gastropods, which differ fundamentally from insects because their bodies consist predominantly of soft, water-rich tissues protected by an external shell. The observed effects of ozone on both soft tissues and shell surface characteristics suggest that ozone can impact the two primary protective components of terrestrial snails, indicating a broad and effective mode of toxic action.

### Limitations of the Study

The present investigation was conducted under controlled laboratory conditions using short-term exposure to relatively high ozone concentrations designed to assess acute toxicological responses. These exposure levels exceed typical ambient environmental concentrations and therefore do not reflect natural field conditions. Accordingly, the findings should be interpreted within the framework of experimental toxicology rather than direct environmental or agricultural application. The study focused primarily on mortality, weight loss, histopathological alterations, and shell surface changes in adult Theba pisana following acute exposure. Chronic exposure scenarios, lower environmentally relevant concentrations, and long-term physiological recovery were not evaluated. In addition, potential effects on non-target organisms, plants, and surrounding environmental components were beyond the scope of this work. Therefore, further investigations under semi-field or field conditions, incorporating environmentally realistic ozone levels and broader ecological assessments, will be necessary to determine environmental safety, reproducibility, and practical feasibility before any consideration of applied use.

## Conclusions

This laboratory-based study evaluated the toxicological effects of ozone gas on the terrestrial snail *T. pisana*, with particular emphasis on mortality, physiological responses, histopathological alterations, and changes in shell surface characteristics. Ozone exposure resulted in concentration-dependent increases in mortality and weight reduction, accompanied by marked tissue damage in the digestive gland and foot, as well as observable modifications in shell surface appearance. These findings demonstrate that ozone can exert measurable toxic effects on both the soft tissues and external protective structures of terrestrial gastropods under controlled conditions.

While the present results provide insight into the biological responses of *T. pisana* to ozone exposure, further investigations are required to clarify the underlying mechanisms of toxicity, define safe and reproducible exposure parameters, and evaluate potential effects on non-target organisms, plants, and surrounding environmental components. Such studies will be essential before considering any practical applications of ozone in agricultural or environmental contexts. In addition, future studies may explore the feasibility of applying ozone under strictly controlled conditions in enclosed systems, such as greenhouses, with careful regulation of concentrations and exposure methods.

## Methods

### Experimental Animals

*Theba pisana*, adult white garden snails, with a shell size about (15–20 mm in diameter)^43^ were collected from infested citrus orchards in El-Raed region, Faisal district, Suez governorate, Egypt. They were identified according to Ali and Ramdane^13^. The gathered snails were transported in plastic bags to the Laboratory of Invertebrates, Faculty of Science, Zoology Department, Al-Azhar University, Cairo, Egypt. Where they were kept in rearing glass boxes measuring 40 × 30 × 30 cm3. These boxes were filled with moist sterilized sandy loamy soil and covered with muslin that was fastened using an elastic band to keep the animals from escape. They were fed daily fresh leaves of lettuce (*Lactuca sativa* L.) for 14 days before treatment for acclimatization. The soil layer was periodically misted with water to maintain the proper humidity and dead snails were removed as quickly as feasible. The snails were collected from privately owned citrus orchards with the verbal consent of the farmers and landowners, who were informed about the purpose of sampling and agreed to the collection procedure.

### The used ozone gas

#### Ozone production

Ozone gas was generated using a device designed by Khaled Hussein Metwaly at the Center of Plasma Technology, Al-Azhar University, Nasr City, Cairo, Egypt. Highly purified extra-dry oxygen (99.9%) was used as the feed gas. Oxygen was introduced into a coaxial dielectric barrier discharge (DBD) reactor, where an alternating high voltage was applied between two electrodes separated by a glass dielectric and an air gap. Under these conditions, filamentary micro-discharges were produced, generating energetic electrons capable of dissociating oxygen molecules (O₂) into atomic oxygen (O). The generated oxygen atoms subsequently reacted with molecular oxygen to form ozone (O₃).

**Fig. 12:**
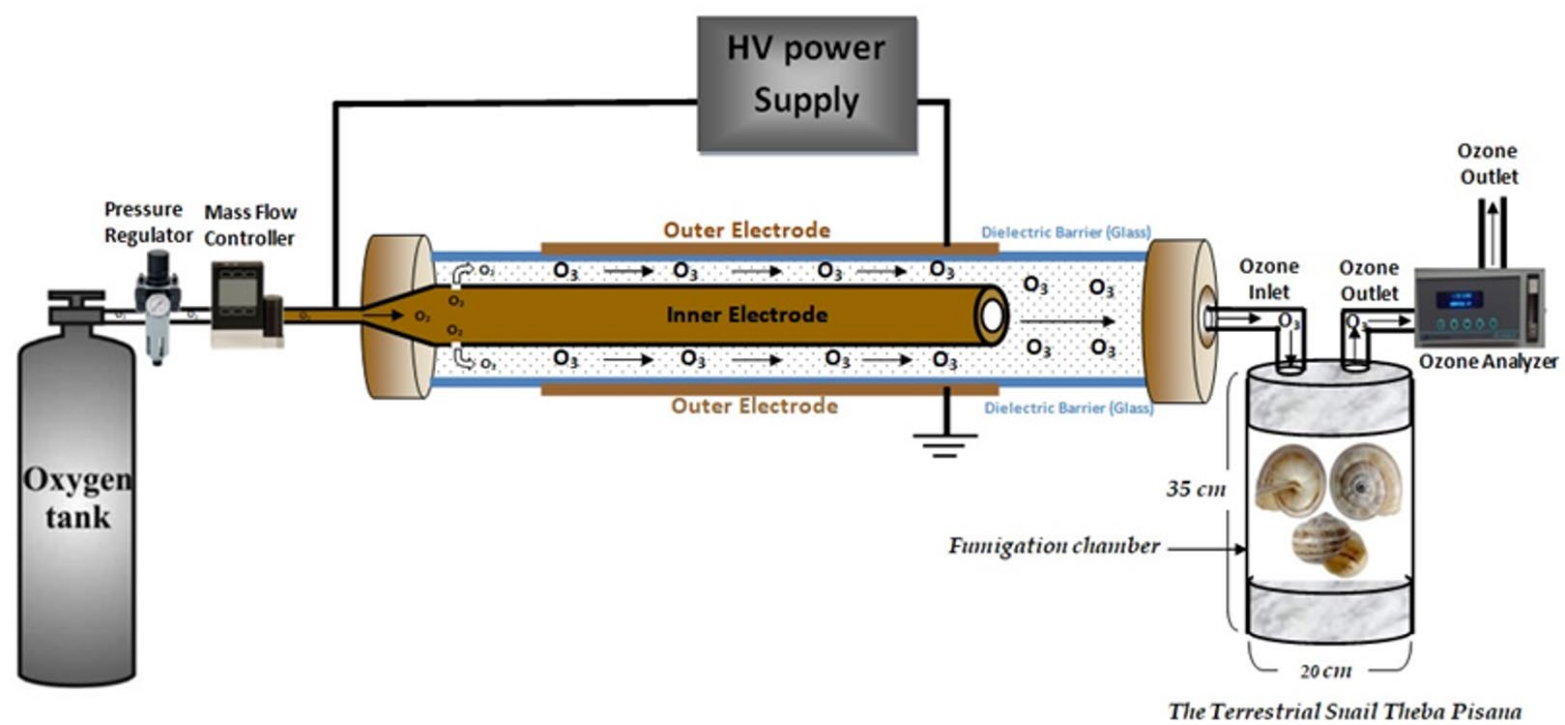
Schematic diagram of ozone generation and fumigation setup.

The dielectric barrier plays a critical role in stabilizing the discharge by limiting excessive current and suppressing arc formation, thereby enabling controlled ozone generation. Ozone concentration inside the exposure chambers was measured using an ozone analyzer (Model H1-AFX, Instrumentation, USA). The AC system operated at an input voltage of 220 V and a frequency of 50 Hz using a high-voltage transformer. The applied voltage was regulated using a Variac variable AC power transformer (Fig. 12). Ozone was produced via silent AC discharge, provided that the electric field strength in the discharge gap exceeded the breakdown threshold of oxygen^44–46^.

1) In pure oxygen, the main reactions that produce oxygen atoms are:

1 $$e+{O_2} \to e+O+O$$

2) Ozone primarily forms through the interaction of oxygen atoms in the following reaction:

2 $$O+{O_2}+M \to {O_3}^{*}+M \to {O_3}+M$$

Where M is a third collision partner (${O}$,${O_2}$,${O_3}$)

At elevated oxygen atom concentrations, competing reactions may occur:

3 $$O+{O_3}+M \to 2{O_2}$$

4 $$O+O_{3}^{*} \to 2{O_2}$$

$O_{3}^{*}$stands for an excited transient ozone species, which is the initial product of reaction.

### Characterization of electrical properties

**Fig. 13:**
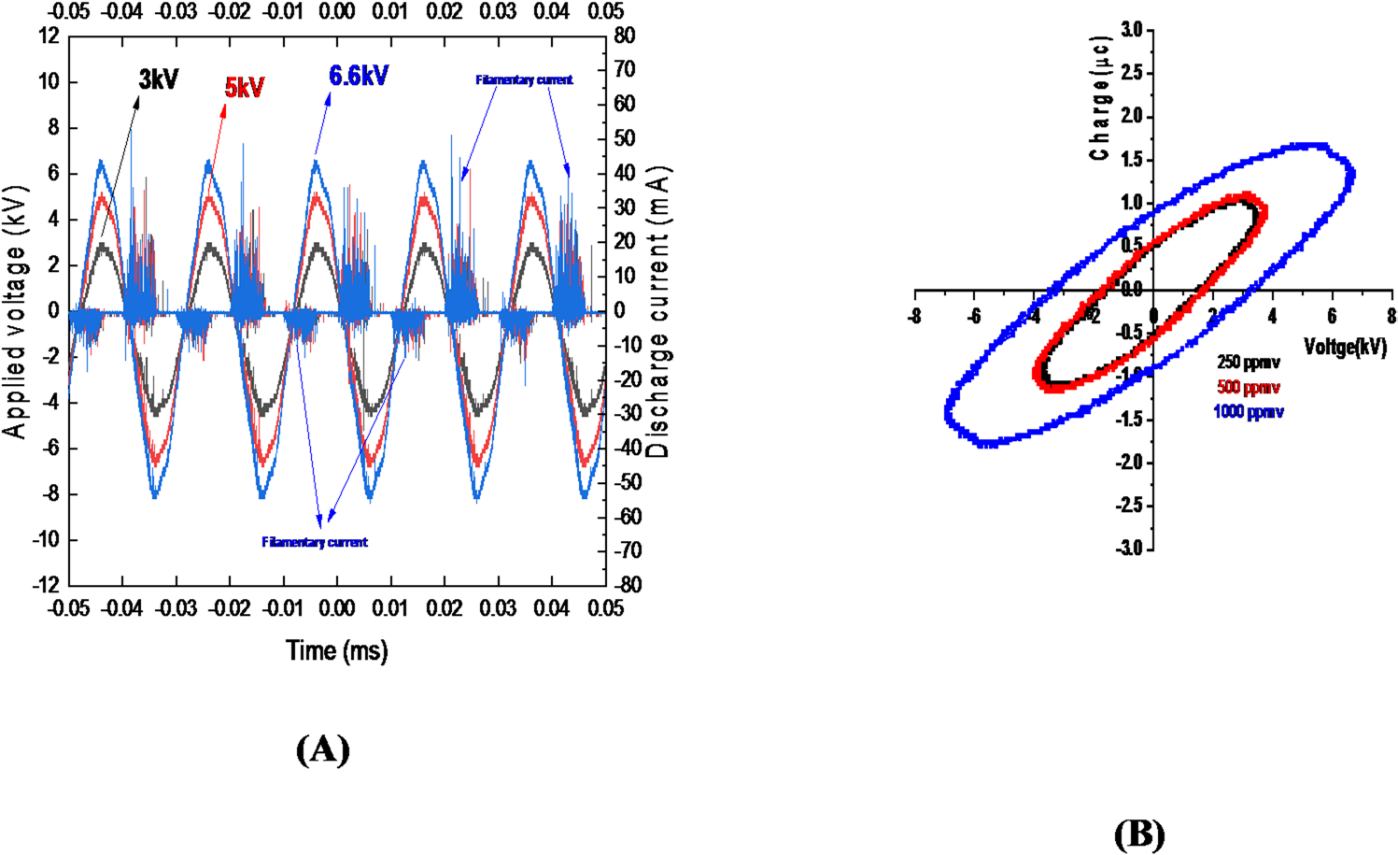
**(A)**: Waveforms of the applied voltage to reactor and associated current measured for Ozone concentration (250, 500, 1000 ppmv) at oxygen flow rate 0.5 (l/min). **(B)**: Lissajous diagrams measured for ozone at flow rate of 0.5 (l/min).

The current–voltage oscillogram of the coaxial dielectric barrier discharge reactor under ambient temperature and atmospheric pressure is shown in Fig. 13A. When the applied voltage exceeded the breakdown voltage, filamentary discharges were observed, resulting in measurable current pulses corresponding to ozone concentrations of 250, 500, and 1000 ppmv (Fig. 13A.). Voltage and current waveforms were recorded at an oxygen flow rate of 0.5 L/min. The corresponding applied voltages were 3, 5, and 6.6 kV, respectively.

### Power measurement method

The discharge power of the coaxial DBD ozone generator was determined using the Lissajous method (Fig. 13B). The energy per voltage cycle (Eₑₗ) was calculated from the area enclosed by the charge–voltage (Q–V) curve. The average electric power (Pₑₗ) was then obtained by multiplying the energy per cycle by the operating frequency (ƒ = 50 Hz):

5 $$P(t)=I(t)V(t)$$

Thus, the input energy per cycle corresponds to twice the area enclosed by the Lissajous figure. This method also allows determination of the minimum ignition voltage (V_min_)^29,40,41,47,48^.

6 $${E_{el}}=2({V_{\hbox{max} }}{Q_{_{0}}} - {Q_{\hbox{max} }}{V_0})={\text{Area of}}{\mkern 1mu} {\text{diagram }}(Q - V){\mkern 1mu} {\mathrm{diagram}}$$

7 $${P_{el}}=\frac{{{E_{el}}}}{T}=f{E_{el}}$$

In this work, the consumed power was measured to be 0.01 W at applied voltage of 3 kV, 0.02 W at 5 kV and 0.1 W at 6.6 kV.

**Fig. 14:**
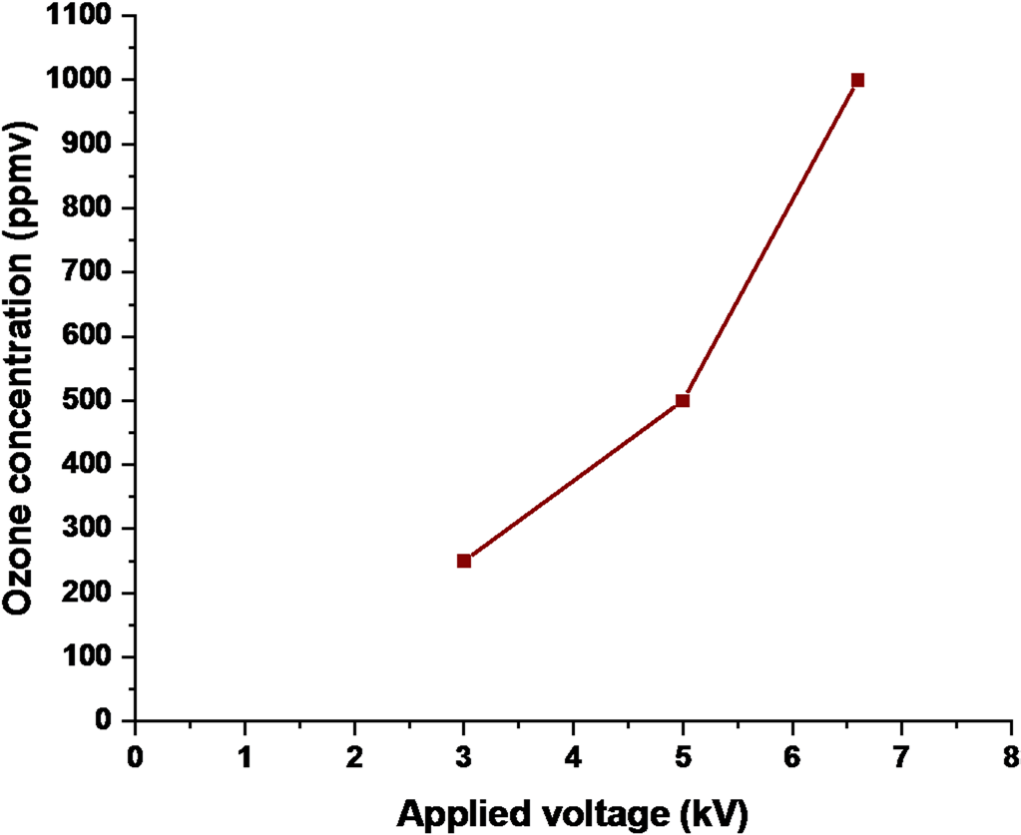
Ozone concentration as a function of applied voltage at 0.5(l/min.) flow rate.

Figure 14 illustrates the relationship between applied voltage and ozone concentration at a constant oxygen flow rate. No ozone was detected below the breakdown voltage. Once the applied voltage exceeded this threshold, ozone concentration increased with increasing voltage. Initially, the increase was gradual, followed by a more rapid rise at higher voltage levels due to enhanced discharge intensity and greater oxygen dissociation efficiency.

### Influence of ozone gas at different concentrations on the adult snails *T. pisana*

Ten adult individuals with approximately similar sizes of the snail *T. pisana* were exposed to ozone gas for half an hour within a fumigation chamber. Three different ozone concentrations (250, 500 and 1000 ppmv) were used, along with a control treatment without ozone treatment. Each treatment was replicated three times (ten adult snails per replicate/thirty per treatment). Post-treatment, the treated groups were transferred to transparent plastic containers (capacity 200 ml each) that were fastened with elastic bands and covered with muslin cloth. The treated groups were examined daily for four days to record and remove dead individuals. Mortality was calculated and corrected using the formula of Abbott^49^. the obtained data were subjected to probit analysis to determine the values ​​of LC_10_, LC_25_, and LC_50_ values after the fourth day using Finney’s probit analysis, per Mekapogu^50^. Regarding the effect of ozone gas on the weight loss of the tested snails, Weight was measured for each replicate (group of ten adult snails) immediately before and 24 h after ozone exposure. Weight loss was calculated as the difference between initial and final weights and expressed both as absolute values (g) and as a percentage of initial weight to account for natural size variation among replicates. The percent weight loss of the snails was estimated following the method given by Jackai and Asante^51^ using the formula Weight loss (%) = IW – FW/IW×100; where IW is the initial weight and FW is the final weight. Weight loss data were subjected to one-way analysis of variance (ANOVA) to evaluate the effect of ozone concentration, including the control group. When significant differences were detected, means were separated using Tukey’s honestly significant difference (HSD) test at *P* < 0.05. These analyses were performed using CoStat software (version 6.311). In addition, a generalized linear model (GLM) with a Gamma distribution and log link function was conducted using SPSS software (version 31 0.0 0.2 0.0 (126)) to further confirm the robustness of treatment effects across concentrations.

### Scanning electron microscope (SEM)

The terrestrial snail *T. pisana* adults treated with different concentrations of ozone were investigated under scanning electron microscope to determine the effect of this gas on the shell surface appearance of the white garden snail. The shells of adult individuals treated and untreated with the tested concentrations were quickly dried, then mounted on specimen stubs with gold conducting paints. Samples were gold coated in a layer of approximately 300A^o^ using a fine gold coating apparatus, ion sputtering device (ZEISS-EVO-15). The untreated and treated samples were examined using a ZEISS-EVO-15 scanning electron microscope (SEM) operating at an accelerating voltage of 20 kV. This work was conducted at the Electron Microscope Unit of the National Center for Radiation Research and Technology (NCRRT), affiliated with the Egyptian Atomic Energy Authority (AEA) in Cairo, Egypt. The dorsal surface of the snail shells was observed and documented through photographs, with the micrographs captured directly from the SEM video monitor.

### Histological investigation

#### Specimen processing and staining

The histopathological investigations were performed on *T. pisana* to show the effect of the ozone gas. *T. pisana* were treated with LC_10_, LC_25_ and LC_50_ of ozone gas and immediately the specimens were fixed after 24 h from the first treatment. The histological investigations were conducted by organizing the study into four groups, with the first group serving as the control. The second group was exposed to LC_10_ of ozone. The third group was exposed to LC_25_ of ozone. The fourth group was exposed to LC_50_ of ozone. Soft tissue from all groups of snails (control and treated snails) was carefully dissected away from the covering outer shell. Collected samples of both the digestive gland and the foot were instantly submerged for 24 h within formalin (10%) for fixation. Fixed samples were dehydrated using ethyl alcohol, after that the samples were cleared using ethyl benzoate, and finally the samples were embedded in paraffin wax. The sections were then cut at a thickness of 4–6 μm. Harris haematoxylin and eosin stain was used to stain the slides^52^. The histopathological investigations of the prepared sections were done by using OLYMPUS BX51 microscope then photographed by using a microscope-adapted OLYMPUSDP72 camera (Department of Anatomy and Embryology, Assiut University’s Faculty of Veterinary Medicine).

## Data Availability

The datasets used and/or analyzed during the current study available from the corresponding author on reasonable request.
